# Quality of Life After Microvascular Alveolar Ridge Reconstruction with Subsequent Dental Rehabilitation

**DOI:** 10.3390/jcm13206229

**Published:** 2024-10-18

**Authors:** Katharina Zeman-Kuhnert, Alexander J. Gaggl, Gian B. Bottini, Joern Wittig, Christoph Steiner, Wanda Lauth, Christian Brandtner

**Affiliations:** 1 Department of Oral and Maxillofacial Surgery, University Hospital of Salzburg, Paracelsus Medical University, 5020 Salzburg, Austria; a.gaggl@salk.at (A.J.G.); g.bottini@salk.at (G.B.B.); j.wittig@salk.at (J.W.); c.steiner@salk.at (C.S.); c.brandtner@salk.at (C.B.); 2 Team Biostatistics and Big Medical Data, IDA Lab Salzburg, Paracelsus Medical University, 5020 Salzburg, Austria; wanda.lauth@pmu.ac.at

**Keywords:** oral health-related quality of life, quality of life, OHIP-49, SF-36, microvascular alveolar ridge reconstruction, dental rehabilitation

## Abstract

**Background/Objectives:** Defects in maxillary and mandibular alveolar ridges are common in maxillofacial practice. Reconstruction with microvascular bone grafts and subsequent prosthetic rehabilitation is the gold standard treatment. This study investigated patients’ quality of life (QoL) after microvascular alveolar ridge reconstruction with subsequent dental rehabilitation. The effect of the underlying disease and success rates of the prosthetic treatment on QoL were analysed. **Methods:** OHIP-49 was used to evaluate oral health-related QoL (OHrQoL). The SF-36 was used to assess disease-nonspecific QoL. **Results:** Fifty-eight patients were enrolled and divided into four diagnostic (malignancy, osteoradionecrosis, benign disease, and cleft palate) and five prosthetic groups (no prosthetics, removable partial dentures, complete dentures, implant-supported removable dentures, and implant-supported fixed dentures). There was a significant difference between the diagnostic groups in the total score of their OHIP-49 (*p* = 0.008). Patients with malignant disease and osteoradionecrosis had worse QoL scores than those with benign diseases and cleft palate. Implant-supported prostheses had the best OHrQoL. Removable partial dentures and patients in whom dental rehabilitation was not possible had the worst OHrQoL (*p* = 0.042). The SF-36 subscale score showed no statistically significant differences between the diagnostic and prosthetic groups (*p* > 0.05). **Conclusions:** OHrQoL after microvascular alveolar ridge reconstruction differs significantly based on underlying diagnoses and prosthetic restorations. Benign diseases and implant-supported dentures have the highest scores.

## 1. Introduction

Defects in maxillary and mandibular alveolar ridges are common in maxillofacial practice. They can occur after trauma, infection, osteonecrosis, congenital jaw deformities, or the surgical resection of benign or malignant tumours. Advanced alveolar ridge defects are often associated with facial disharmony, impairments in speech, or impaired mastication and dietary limitations [1,2,3,4]. Reconstruction with microvascular bone grafts and subsequent prosthetic rehabilitation is the favoured choice of treatment for these defects [5,6,7].

However, this is a complex process and must be tailored to the underlying disease and anatomical conditions of the reconstructed jaw and mucosa. Complications during both treatment and subsequent prosthetic restoration can have a major functional and emotional impact on patients [1,8,9]. As a result, the quality of life (QoL) often suffers in these patients.

Studies have demonstrated that patients with oral and maxillofacial pathologies exhibit worse QoL scores compared to healthy controls. Patients with head and neck cancers have poor oral function and, hence, low scores in the psychological domain [10,11,12]. Patients with cleft lip and palate are more likely to have decreased self-esteem, greater difficulty in social interactions [13], and physical and social disabilities due to poor oral health [14].

To the best of our knowledge, no previous studies have assessed the QoL of patients with extended alveolar ridge reconstructions, regardless of the underlying cause or prosthetic outcome. Assuming that both the underlying diagnosis and the outcome of prosthetic treatment influence QoL, our study population was investigated from both aspects. This study assessed the extent to which the underlying disease and varying success rates of dental rehabilitation influenced oral and overall QoL after microvascular alveolar ridge reconstruction.

## 2. Materials and Methods

### 2.1. Patients

We retrospectively searched the University Hospital Salzburg’s database to identify all the patients who had undergone extended segmental alveolar ridge reconstruction with a microvascular free flap at the Department of Oral and Maxillofacial Surgery between January 2011 and December 2018. Patients aged >18 years were invited to participate in a QoL survey between June 2020 and July 2021. All participants provided written informed consent prior to their enrolment in the study.

The medical records of all study participants were reviewed to record patient age and sex, indications for microvascular segmental alveolar ridge reconstruction, months between the surgery and survey, anatomical resection and reconstruction site, type of microvascular flap, and type of dental rehabilitation (none, removable prosthesis (complete/partial), or implant-supported (removable/fixed)).

### 2.2. Assessment Instruments

QoL was evaluated using two previously validated questionnaires. The Oral Health Impact Profile 49 (OHIP-49) is used to assess the impact of oral conditions on oral health-related quality of Life (OHrQoL) [15]. It consists of 49 items, which are grouped into seven topics: ‘functional limitations’, ‘physical pain’, ‘psychological discomfort’, ‘physical disability’, ‘psychological disability’, ‘social disability’, and ‘handicap’. The score of all seven topics added together results in a total OHIP-49 score between 0 and 196 (0 = best OHrQoL).

The 36-item Short Form Health Survey (SF-36) is a disease-nonspecific questionnaire used to assess QoL [16,17]. It was developed from the Medical Outcomes Study and is primarily used to evaluate the benefits of medical therapies [18]. Its 36 items are related to physical, psychological, and social dimensions. They can be combined into eight subscales that represent subjective health experience. For each subscale, the scores range from 0 to 100 (100 = best quality of life).

In addition to the questionnaires, further questions were asked:

(1) Have you been able to engage in your daily business (work or study) since your diagnosis?
  - Yes, or I am retired (age-related).
  - No, I took early-retirement/am occupationally disabled due to illness.

(2) Do you have any other diseases or physical, social, or psychological complaints that are affecting your QoL?

### 2.3. Statistical Analysis

In order to analyse the data, relationships were visualised and represented by counts, medians, interquartile ranges, and Spearman’s correlation coefficients to show monotonic correlations. Nonparametric models were used to assess differences between groups using the R package rankFD [19]. This was because of the small and unbalanced group sizes, potential ‘outliers’, and the ordinal scale of the QoL scores. For these nonparametric ANOVA-type statistics, a two-sided significance level of alpha = 0.05 was considered significant for each test. Group comparisons were carried out for the whole cohort regarding the overall OHIP-49 score and SF-36 scores for sex, jaw, or flap type. The diagnostic and prosthodontic groups were statistically compared in terms of their overall OHIP-49 score and its subscales, as well as their SF-36 scores. The Bonferroni–Holm method was used to adjust for multiplicity. Statistical analyses were conducted using the statistical software R (version 4.1.3) [20].

## 3. Results

A total of 207 patients underwent alveolar ridge reconstruction at the University Hospital Salzburg between January 2011 and December 2018. Of these, 149 survived the study period. Seven patients were excluded because of loss to follow-up. Of the remaining 142 patients, 58 agreed to complete the questionnaires. Thirty-three patients were male (57%), and the mean age at the time of the survey was 59.6 years (range 22–88 years). The indications for microvascular alveolar ridge reconstruction included malignant tumours (41.4%, n = 24), benign diseases (34.5%, n = 20), cleft palate (15.5%, n = 9), and osteoradionecrosis (8.6%, n = 5). The prosthetic care of the total study population, as well as within the individual diagnostic groups, is demonstrated in Table 1.

In 53.4% of cases, the maxilla was the reconstructed jaw. In 16 patients (27.6%), a bone graft with a soft-tissue paddle was used. On average, 62 months (range 15–122 months) had elapsed between the reconstruction and the survey.

Eleven patients (19%) were incapacitated/retired early because of underlying disease at the time of the study.

### 3.1. Oral Health-Related Quality of Life

The median overall OHIP-49 score was 13.5 (IQR 3.25–36.75; range 0–115) for the entire study population. The overall OHIP-49 score for the cohort demonstrated no significant association with sex (*p* = 0.519), jaw (maxilla or mandible) (*p* = 0.173), or flap type (bone flap with or without a soft-tissue paddle) (*p* = 0.487). We observed no significant correlations between overall OHIP-49 score and age (ρ = −0.023) or the time between surgery and the survey (ρ = −0.019).

#### 3.1.1. Diagnostic Groups

**Figure 1:**
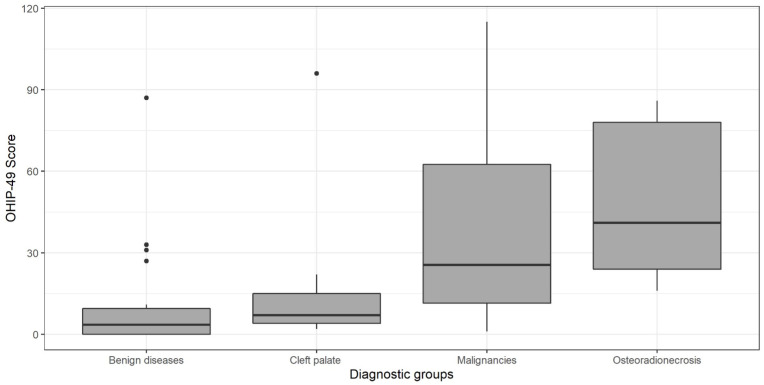
OHIP-49 scores for diagnostic groups.

The median overall OHIP-49 score was 25.5 for malignancies (IQR 11.5–62.5), 3.5 for benign diseases (IQR 0–9.5), 7.0 for cleft palates (IQR 4–15), and 41.0 for osteoradionecrosis (IQR 24–78) (Figure 1). The difference in overall OHIP-49 scores between the diagnostic groups was statistically significant (*p* = 0.008).

The ‘benign disease’ and ‘cleft palate’ diagnosis groups each included one patient with a much higher OHIP-49 score than the average (benign disease score = 87; cleft palate score = 96).

There were statistically significant differences between the four diagnostic groups in all OHIP-49 subscales except for ‘handicap’, ‘psychological disability’, and ‘social disability’ (Table 2). We observed no statistically significant differences in the ability to work among the four diagnostic groups (*p* = 0.463).

#### 3.1.2. Prosthodontic Groups

The study populations were compared regarding their dental rehabilitation (Table 3). The two groups of implant-supported prosthodontics demonstrated the best OHrQoL, followed by the complete denture group and the group with no prosthodontics. The OHrQoL score of the partial removable denture group was the worst. This difference was statistically significant (*p* = 0.042). When combining both conventional removable denture groups into one group to adjust the group sizes, the difference remained significant (*p* = 0.048).

Again, as previously mentioned for the diagnostic groups, the same two patients stood out as outliers because of their high OHIP-49 scores.

All OHIP-49 subscale scores indicated statistically significant (*p* < 0.05) differences between the five prosthodontic groups. Here, too, the differences in the subscale values remained statistically significant when the ‘partial removable denture’ and ‘complete denture’ groups were combined into one group (*p* < 0.05). No statistically significant differences were observed between the prosthodontic groups in terms of their work ability (*p* = 0.102).

### 3.2. Disease Non-Specific Quality of Life

The median SF-36 subscale scores were between 72.5 and 100 out of the possible 100 points for the total cohort (Table 4).

No subscale exhibited significant association with sex, jaw (maxilla or mandible), or flap type (bone flap with or without a soft-tissue paddle) (*p* > 0.05).

Only the subscale ‘role limitation due to physical health’ (r = 0.210) demonstrated a small correlation with age. There was no correlation between each subscale and the variable ‘time between surgery and survey’ (r ≤ 0.1).

#### 3.2.1. Diagnostic Groups

Table 5 presents the medians and IQR of the SF-36 subscale scores for the four diagnosis groups. The cleft palate group had the highest values for self-perceived QoL in all subscales among all diagnostic groups.

There were no statistically significant differences between the four diagnostic groups in all subscales (*p* > 0.05).

Two patients demonstrated consistently poor scores in all subscales.

**Figure 2:**
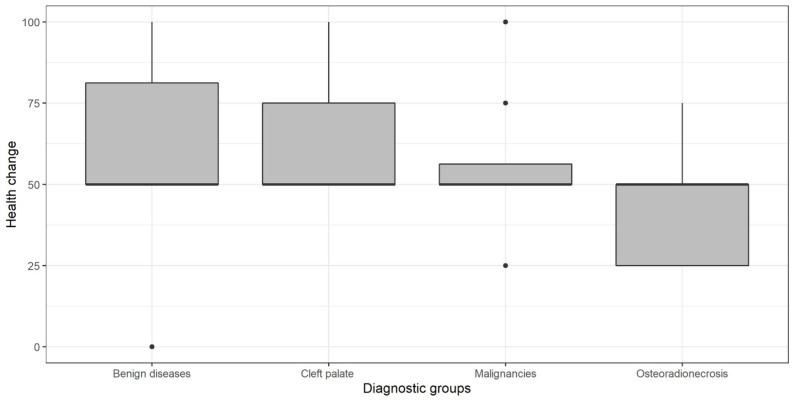
SF-36 subscale ‘health change’ for diagnostic groups.

When asked if their health status had remained the same (score = 50), improved (score > 50), or worsened (score < 50) compared with the previous year, only the group with osteoradionecrosis reported a decrease in health status (Figure 2).

None of the eight SF-36 subscales within the diagnostic groups were negatively affected by an incapacity to work or early retirement due to the underlying disease in terms of their quality of life (*p* > 0.05).

**Figure 3:**
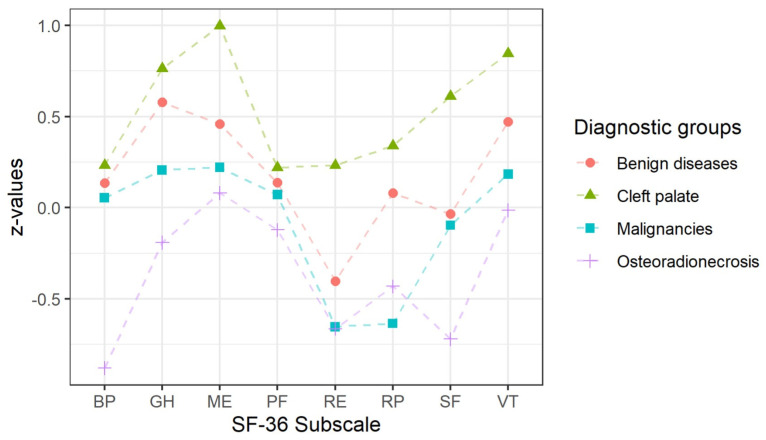
SF-36 subscales—deviations from the German norm population [17]. Abbreviations: BP, bodily pain; GH, general health; ME, mental health; PF, physical functioning; RE, role limitation due to emotional problems; RP, role limitation due to physical health; SF, social functioning; VT, vitality.

In Figure 3, the deviation of the SF-36 subscale score values from the standardised norm population can be observed for each subgroup [16,17].

#### 3.2.2. Prosthodontic Groups

The medians and IQR of the SF-36 subscales for the five prosthodontic groups are presented in Table 6. Patients with conventional partial dentures had the lowest median scores for all the subscales. Patients with implant-supported dentures (both removable and fixed) had the best QoL scores for all subscales. No significant differences in all eight subscale scores were observed between the five prosthodontic groups (*p* > 0.05; Table 6). Even when the ‘partial removable denture’ and ‘complete denture’ groups were combined into one group to adjust the group sizes, no statistically significant difference was found between the prosthetic groups in any subscale score (*p* > 0.05; Table 7).

Again, the same two patients stood out as outliers due to their worse QoL results across all subscales compared to their group mates.

When considering early retirement/unfitness for work due to disease, there was a statistical significance in the subscale ‘social functioning’ (*p* = 0.025). None of the other subscales demonstrated statistical significance with regard to this cofactor (*p* > 0.05).

### 3.3. Correlation of OHIP-49 and Short Form-36

**Figure 4:**
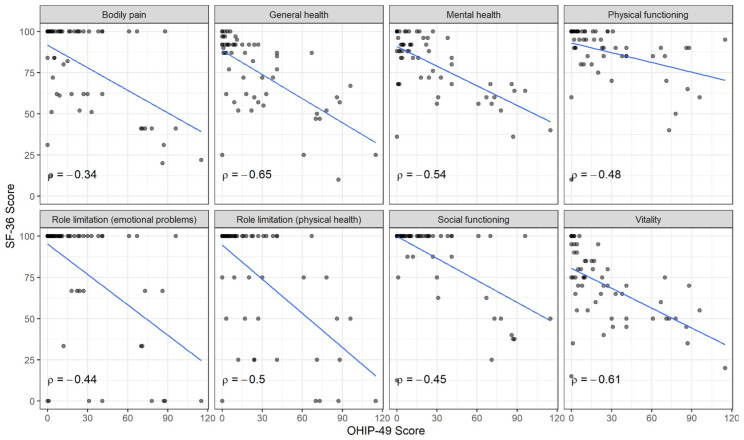
Correlation between OHIP-49 scores and SF-36 subscale scores.

We observed a correlation between all SF-36 subscales and the total OHIP-49 score (Figure 4). The strongest correlation was demonstrated by the subscales ‘general health’ (ρ = −0.650), ‘vitality’ (ρ = −0.610), and ‘mental health’ (ρ = −0.540).

## 4. Discussion

We assessed the OHrQoL and disease-nonspecific QoL in patients who underwent microvascular alveolar ridge reconstruction and classified them according to both their underlying diagnosis and prosthetic outcome.

### 4.1. Diagnostic Groups

Among the diagnostic groups, the benign disease group demonstrated the best OHrQoL, followed by the cleft palate group. This can be explained by the fact that only smaller areas of the alveolar ridge were affected, and patients were routinely provided with implant-supported dentures.

The group with osteoradionecrosis demonstrated the worst OHrQoL, which was similar to the findings reported by Jacobson et al. (2013) in their study on QoL after osteoradionecrosis management. They observed that QoL scores on the ‘Eating Assessment Tool—10’ and the ‘Speech Handicap Index’ were worse for the osteoradionecrosis group compared to a control group [22]. On the SF-36 questionnaire, the osteoradionecrosis group exhibited the worst ‘general health’ scores. It is also known from the literature that the morbidity of patients with advanced osteoradionecrosis is comparable to or even worse than those treated for advanced cancer [23].

The SF-36 subscale ‘mental health’ demonstrated lower scores in the malignancy group. Patients with malignant diseases consider themselves more stressed because of strenuous therapies, sadness, sleep problems, and increased anxiety levels [24,25].

When comparing the SF-36 study results with a standardised norm population [16,17], patients with cleft palate demonstrated better QoL values on all subscales, while those with osteoradionecrosis demonstrated worse values than the norm population. However, drawing definite conclusions was difficult because of the limited sample size. Overall, the QoL of our study population measured by the SF-36 might be considered quite similar to that of the norm population (Figure 3).

### 4.2. Prosthodontic Groups

Considering the outcomes of prosthetic restoration, it was observed that the best OHrQoL was associated with implant-supported fixed prostheses and the worst with removable partial dentures, followed by patients without dental rehabilitation. Patients with conventional partial dentures and those for whom dental rehabilitation was not possible demonstrated the lowest QoL scores. The poor QoL outcomes in both questionnaires are understandable for patients without prosthodontics, as speech, eating, and aesthetics are compromised by edentulism [26].

However, the results for conventional partial dentures appear surprising. The conventional removable prosthesis group was small, as it only included three patients. Therefore, it can be assumed that this result could be due to individual phenomena. All three patients had oral cancer as an underlying disease. Of these, two patients had a secondary tumour. One patient had a prostate carcinoma that was irradiated at the time of the survey. The other patient underwent esophagectomy in 2017 for squamous cell carcinoma. Since then, their food intake has occurred exclusively through the jejunocaths. The third patient reported severe frontal headaches radiating to the face and upper jaw at the time of the survey. The patient was referred to the neurology department for evaluation.

Furthermore, a previous study investigating the change in OHrQoL following prosthetic treatment also demonstrated the least improvement in patients treated with removable partial dentures compared to those treated with implant-supported dentures [27].

A comparison of our OHrQoL results with the German OHIP-49 norm values reported for different tooth replacement groups was encouraging [21]. Our population demonstrated better values in the complete and implant-supported removable denture groups than those in the German comparison groups. The group with implant-supported fixed prostheses demonstrated a slightly worse OHrQoL; however, the comparison group also included completely dentate patients (Table 3). Due to the small number of study participants with partial dentures (n = 3), a comparison with the norm population is not meaningful, as the scores are more likely to be due to individual characteristics.

### 4.3. Outliers and Further Observations

Within our study population, three outliers scored poorly compared with their corresponding group members. One patient scored the lowest on both QoL questionnaires, although receiving alveolar ridge reconstruction because of a benign disease. The alveolar ridge was restored with an implant-supported removable prosthesis. However, just a few weeks before the survey, the patient underwent surgery to remove a fistula caused by an infected implant in the reconstructed jaw. The patient recovered and their complaints subsided completely. The second outlier, a patient with a cleft palate and complete denture, reported worse OHIP-49 scores than their respective group members. However, this patient had an implant and had undergone vestibuloplasty within the month of the survey. Therefore, the patients’ OHrQoL was affected by the presence of surgical wounds. The third patient demonstrated very poor SF-36 scores, whereas their OHIP-49 score was good. The alveolar ridge reconstruction was for a benign disease, and the patient was very satisfied with their implant-supported removable denture. However, at the time of the study, the patient had progressive lung cancer that affected the patient’s physical and mental health.

We observed that an incapacity to work/early retirement due to the underlying disease did not negatively influence OHIP-49 or SF-36 scores. Therefore, bias in our results due to the subjects’ inability to cope with normal daily life, as detected in other studies, can be excluded [28,29].

We also observed a correlation between the OHIP-49 total score and all SF-36 subscales. This correlation was most robust in the ‘general health’ (ρ = −0.650), ‘vitality’ (ρ = −0.610), and ‘mental health’ (ρ = −0.540) domains. Therefore, it can be concluded that oral health has an impact on overall physical and mental quality of life.

A limitation of the survey was the sample size of only 58 participants and the unequal group sizes, especially in the prosthodontic groups. Therefore, the statistics for both the OHIP-49 and SF-36 scores were recalculated with adjusted group sizes by combining the two conventional removable groups (partial and complete) into one. Interestingly, the same statistical results were observed for OHrQoL and overall disease-nonspecific quality of life as those without combining the groups. Therefore, the data are representative enough to illustrate the impact of alveolar reconstruction on patients’ quality of life.

## 5. Conclusions

Patients’ QoL after segmental alveolar ridge reconstruction differs significantly in terms of their underlying diagnoses and prosthetic restorations. Patients with benign diseases and those who underwent restoration with implant-supported fixed prostheses had the highest OHrQoL scores. In contrast, patients with osteoradionecrosis or partial dentures, or those without dental rehabilitation, had the poorest OHrQoL.

## Figures and Tables

**Table 1:** Outcomes of dental rehabilitation within diagnostic subgroups.

	No Denture	Partial Denture	Complete Dentures	Implant-Supported Removable Denture	Implant-Supported Fixed Denture
Malignancies	10	3	3	6	2
Benign diseases	0	0	1	10	9
Cleft palate	0	0	2	3	4
Osteoradionecrosis	3	0	0	2	0

**Table 2:** OHIP-49 subscale medians (IQR) for the diagnostic groups.

OHIP Subscale	Malignancies	Benign Diseases	Cleft Palate	Osteoradionecrosis	*p*-Value
Functional limitation	7.5 (2–13)	1 (0–2.25)	3 (2–6)	9 (8–15)	0.013
Handicap	2 (0–7.25)	0 (0–0)	0 (0–0)	4 (2–9)	0.126
Physical pain	2.5 (0.75–7.25)	1.5 (0–4)	2 (0–4)	11 (6–13)	0.008
Physical disability	6 (2.75–12.25)	0 (0–0.5)	2 (1–4)	4 (3–15)	0.043
Psychological disability	2 (0–5)	0 (0–1.25)	0 (0–0)	7 (1–7)	0.126
Psychological discomfort	2.5 (0–7)	0 (0–2)	0 (0–3)	6 (4–10)	0.002
Social disability	0.5 (0–5)	0 (0–0)	0 (0–0)	2 (0–6)	0.204

**Table 3:** OHIP-49 score percentiles for the prosthodontic groups and German OHIP-49 norm values [21].

	Prosthodontics	30th Percentile	Median	70th Percentile
Study population	No dental rehabilitation	21.8	38	73.8
	Partial removable denture	68.8	70	71.2
	Complete denture	13	25.5	34
	Implant-supported removable denture	4	7	12
	Implant-supported fixed denture	2.2	8	14
German norm values	Without denture (fully dentate, fixed prosthodontics)	1	5	13
	Removable denture	8	15	31
	Complete denture	6	23	45

**Table 4:** SF-36 subscale medians and interquartile range—total cohort.

SF-36 Subscale	Median	Interquartile Range
Physical functioning	92.5	85–100
Role limitation (physical health)	100	50–100
Role limitation (emotional problems)	100	66.7–100
Vitality	72.5	55–85
Mental health	88	68–95
Social functioning	100	87.5–100
Bodily pain	100	62–100
General health	87	60–92

**Table 5:** SF-36 subscale medians (IQR) for the diagnostic groups.

SF-36 Subscale	Malignancies	Benign Diseases	Cleft Palate	Osteoradionecrosis	*p*-Value
Physical functioning	90 (85–95)	100 (90–100)	100 (80–100)	90 (85–90)	0.800
Role limitation due to physical health	75 (25–100)	100 (100–100)	100 (100–100)	75 (50–100)	0.690
Role limitation due to emotional problems	100 (58.35–100)	100 (100–100)	100 (100–100)	100 (85–90)	0.795
Vitality	67.5 (50–81.25)	75 (62.5–96.25)	80 (70–90)	70 (50–70)	0.690
Mental health	76 (63–96)	88 (71–94)	92 (88–100)	84 (68–84)	0.302
Social functioning	100 (84.4–100)	100 (87.5–100)	100 (100–100)	87.5 (50–100)	0.690
Bodily pain	100 (62–100)	100 (61.75–100)	100 (82–100)	52 (41–62)	0.690
General health	83.5 (55.75–90.5)	92 (62–97)	92 (77–92)	60 (60–72)	0.082

**Table 6:** SF-36 subscale medians (IQR) for five prosthodontic groups.

SF-36 Subscale	No Dental Rehabilitation	Partial Denture	Complete Denture	Implant-Supported Removable Denture	Implant-Supported Fixed Denture	*p*-Value
Physical functioning	90 (85–90)	85 (62.5–87.5)	95 (86.25–100)	100 (85–100)	95 (85–100)	0.941
Role limitation due to physical health	75 (25–100)	0 (0–50)	75 (50–100)	100 (75–100)	100 (100–100)	1.000
Role limitation due to emotional problems	100 (33.3–100)	66.7 (50–83.35)	100 (75–100)	100 (33.3–100)	100 (100–100)	1.000
Vitality	70 (50–85)	60 (55–67.5)	67.5 (47.5–100)	75 (55–85)	75 (67.5–92.5)	1.000
Mental health	68 (56–96)	60 (60–64)	86 (69–100)	88 (80–92)	88 (80–96)	0.158
Social functioning	100 (50–100)	62.5 (56.25–81.25)	100 (100–100)	100 (87.5–100)	100 (100–100)	1.000
Bodily pain	100 (41–100)	41 (41–70.5)	100 (100–100)	100 (62–100)	84 (62–100)	1.000
General health	60 (52–87)	47 (47–67)	89.5 (72–98)	87 (62–92)	92 (74.5–94.5)	0.731

**Table 7:** SF-36 subscale medians (IQR) for four prosthodontic groups.

SF-36 Subscale	No Dental Rehabilitation	Removable Denture (Partial and Complete)	Implant-Supported Removable Denture	Implant-Supported Fixed Denture	*p*-Value
Physical functioning	90 (85–90)	90 (85–100)	100 (85–100)	95 (85–100)	1.000
Role limitation due to physical health	75 (25–100)	90 (85–100)	100 (75–100)	100 (100–100)	0.269
Role limitation due to emotional problems	100 (33.3–100)	100 (66.7–100)	100 (33.3–100)	100 (100–100)	1.000
Vitality	70 (50–85)	60 (50–80)	75 (55–85)	75 (67.5–92.5)	1.000
Mental health	68 (56–96)	68 (64–100)	88 (80–92)	88 (80–96)	1.000
Social functioning	100 (50–100)	100 (100–100)	100 (87.5–100)	100 (100–100)	1.000
Bodily pain	100 (41–100)	100 (41–100)	100 (62–100)	84 (62–100)	1.000
General health	60 (52–87)	87 (57–92)	87 (62–92)	92 (74.5–94.5)	0.988

## Data Availability

The data presented in this study are available on request from the corresponding author.
